# Ability of a biomarker-based score to predict death from circulatory disease and cancer in NHANES III

**DOI:** 10.1186/1471-2458-12-895

**Published:** 2012-10-23

**Authors:** Mieke Van Hemelrijck, Monika Eichholzer, David Faeh, Sabine Rohrmann

**Affiliations:** 1 King’s College London, School of Medicine, Division of Cancer Studies, Cancer Epidemiology Group, Research Oncology, 3rd Floor, Bermondsey Wing, Guy’s Hospital, London, SE1 9RT, UK; 2 Institute of Social and Preventive Medicine, University of Zurich, Zurich, Switzerland

**Keywords:** Mortality, Albumin, HDL-cholesterol, C-reactive protein, Gamma-glutamyltransferase

## Abstract

**Background:**

A score based on serum concentrations of C-reactive protein (CRP), albumin, gamma-glutamyl transferase (GGT), and HDL cholesterol was positively associated with death from cancer, circulatory disease, and all-cause mortality. We replicated this in the third National Health and Nutrition Examination Survey (NHANES III), a US nationally representative survey conducted between 1988–1994.

**Methods:**

Baseline measurements of CRP, albumin, GGT, and HDL were available for participants with mortality follow-up (n=13,056). A biomarker score, ranging 0–4, was created by adding number of markers with abnormal values (cut-off: CRP>10mg/L, albumin<35mg/L, GGT>36U/L, HDL<1.04mmol/L). Its association with mortality was analyzed with multivariate Cox proportional hazards models.

**Results:**

The score was positively associated with death from all causes, cancer and circulatory disease [e.g. HR all-cause mortality: 1.21 (95% CI: 1.09, 1.35), 1.92 (1.67, 2.20), 3.38 (2.62, 4.36), and 7.93 (5.77, 10.89), for score 1, 2, 3, 4 vs.0]. These patterns were found across the Charlson Comorbidity Index (CCI). Where CCI =3, risk of cancer death was 1.09 (0.93, 1.28), 1.81 (1.43, 2.29), 4.67 (3.05, 7.14), and 6.97 (5.32, 9.14) for score 1, 2, 3, 4 vs. 0. No effect-modification by sex or race/ethnicity was observed.

**Conclusions:**

These findings correlate with results from a Swedish study. This biomarker-based score could help clinicians make decisions in prevention and disease management.

## Background

In a large prospective cohort study from Sweden, the combination of easily and inexpensively measurable biomarkers clearly predicted mortality in persons aged 50+ years [1]. This mortality score included serum levels of C-reactive protein (CRP), albumin, gamma-glutamyltransferase (GGT), and high-density lipoprotein (HDL) cholesterol, all reflecting possible mechanisms contributing to early death: inflammation [2,3], hepatic function [4], and lipid metabolism [5,6]. The score was positively and progressively associated with all-cause mortality as well as cancer and circulatory disease-specific death. Even among cancer patients with no other co-morbidities, according to the Charlson Comorbidity Index (CCI), the score was predictive for mortality [1]. The mortality score’s ability to predict death is important, especially given the need for an assessment of functional rather than chronological age when treating cancer patients, as recently expressed by the European Organization for Research and Treatment of Cancer (EORTC) Elderly Task Force [7]. Following the rising life expectancy in many countries, it is of importance to accurately predict cancer burden among aging cancer populations suffering from concomitant disease at time of cancer diagnosis.

We examined the association of this biomarker-based score with mortality and with respect to CCI and other potential modifying factors among 13,056 participants in the Third National Health and Nutrition Examination Survey (NHANES III), a large study designed to be nationally representative of the United States population.

## Methods

### Study population and data collection

This study is based on the NHANES III Mortality linkage which provides follow-up data from the date of NHANES III survey participation (1988–1994) through December 31, 2006.

NHANES III is a cross-sectional study conducted by the National Center for Health Statistics (NCHS) between 1988 and 1994 [8]. It was designed as a multistage stratified, clustered probability sample of the US civilian non-institutionalized population at least two months old. Subjects participated in an interview conducted at home and an extensive physical examination, which included a blood sample, conducted in a mobile examination center [8]. NHANES III was conducted in two phases (1988–1991 and 1991–1994). Unbiased national estimates of health and nutrition characteristics can be independently produced for each phase. Within each phase, subjects were randomly assigned to participate in either the morning or afternoon/evening examination session. In total, 30,818 people were interviewed in NHANES III and had a physical examination and a blood sample taken. The NCHS has updated the mortality linkage of NHANES III to death certificate data found in the National Death Index (NDI) so that mortality ascertainment is based upon the results from a probabilistic match between NHANES III and NDI death certificate records [9]. From this linkage, we selected all men and women eligible for mortality follow-up (all participants 17 years of older) with baseline measurements of CRP, GGT, HDL, and albumin (n=13,056).

Serum CRP was quantified using latex-enhanced nephelometry, standardized according to the World Health Organization's international reference preparation of CRP with the coefficient of variation ranging from 3.2–16.1% (median, 6.3%) through the study period [10]. Serum GGT concentration was assayed with a Hitachi 737 Analyzer (Boehringer–Mannheim Diagnostics, Indianapolis, IN, USA) with coefficients of variation ranging from 1.9 to 3.5% [10]. HDL cholesterol was measured using standard enzymatic methods and serum albumin was assessed with the bromcresol purple method. Details about all laboratory procedures have been published elsewhere [10].

Other covariates in the analysis included age, race/ethnicity, poverty-to-income-ratio, comorbidity index, smoking, alcohol consumption, and physical activity. Race and ethnicity were combined into four race–ethnic groups: Non-Hispanic white, Non-Hispanic black, Mexican American and Other. The poverty to income ratio is an index of poverty status which is calculated by dividing family income by a poverty threshold specific to family size.

Comorbidity was evaluated with a comorbidity coefficient similar to the CCI, as used in other NHANES III-based analyses [11]. Each of the comorbidities available in the dataset contributed one point to the composite index with additional points given for older age. Cigarette smoking, alcohol consumption, and physical activity were assessed using questionnaires. The protocols for the conduct of NHANES III were approved by the institutional review board of the National Center for Health Statistics, Centers for Disease Control and Prevention. Written informed consent was obtained from all participants.

### Data analysis

To address the combined association between the four biomarkers of interest and all-cause mortality we started with a multivariate Cox proportional hazards regression using continuous variables of CRP (mg/L), albumin (g/L), GGT (U/L), and HDL (mmol/L). A mortality score, ranging from 0 to 4, was then calculated as the number of biomarkers with abnormal values according to their clinical cut-offs (CRP>10mg/L, albumin<35g/L, GGT>35U/L, and HDL<1.03mmol/L) [3,12,13]. Multivariate Cox proportional hazards regression was used to investigate this mortality score in relation to all-cause mortality as well as cancer-specific (ICD10:C00-C99) and circulatory-specific death (ICD10: I00-I99). Sampling weights for NHANES III were used to account for sampling variability and to adjust for differential probability of selection of persons [8]. All models were adjusted for age (continuous), race/ethnicity, poverty to income ratio, comorbidity index, smoking, alcohol consumption, physical activity, and BMI. A test for trend was conducted by using assignment to quartiles as an ordinal scale, meaning a continuous variable ranging from 1 to 4 indicating the different quartiles. Stratified analyses were conducted for men and women [14], age-groups (<65 and 65+), different categories of the CCI, and ethnic groups. The latter offers the opportunity to evaluate whether the score holds across different ethnic groups and is in concordance with many other studies performed in NHANES [15-17]. We also assessed the association between the mortality score and the CCI by calculating the correlation coefficient and kappa’s coefficient of agreement between both measurements. All tests were two-sided; p-values <0.05 were considered to be statistically significant. All analyses were conducted with Statistical Analysis Systems (SAS) release 9.1.3 (SAS Institute, Cary, NC).

## Results

A total of 3,081 persons died during follow-up time, of whom 653 (21.19 %) died of cancer and 1,388 (45.05%) of circulatory disease. Population characteristics are shown in Table 1. A higher CCI was observed for those who died during follow-up than for those who were alive at the end of follow-up (32.91% versus 8.11% with CCI=4+) (Table 1).

**Table 1:** Descriptive statistics of study population by vital status

	**Survivors** (**N**=**9**,**975**)	**Deaths from all causes** (**N**=**3**,**081**)
**Mean Age** (years) (SE)	39.14 (0.31)	65.53 (0.55)
**Gender**		
Men	47.48	49.50
Women	52.52	50.50
**Mean follow**-**up time** (months) (SE)	174.14 (2.60)	95.17 (1.91)
**Race** - **Ethnicity**		
Non-Hispanic white	74.40	80.71
Non-Hispanic black	10.98	11.99
Mexican American	5.33	2.53
Other	9.28	4.78
**Poverty to income ratio**		
<1.4	18.66	25.98
1.4-3.17	33.47	37.10
≥3.17	47.87	36.92
**Comorbidity Index** (**CCI**)		
0	0.43	0.08
2	69.22	32.32
3	22.85	35.05
4+	7.51	32.55
**Alcohol consumption**		
Never	42.54	62.34
Up to once a week	20.27	13.77
2-3 times a week	15.27	6.70
4-6 times a week	12.33	6.47
Daily or more	9.59	10.72
**Smoking behaviour**		
Never	49.59	37.49
Former	22.57	35.68
Current	27.84	26.83
**Vigorous Physical activity**	8.44	24.91
**BMI** (kg/m^2^)		
<18.50	2.53	3.37
18.50-24.99	44.38	35.16
25.00-29.99	31.71	35.67
>30.00	21.38	25.80
**CRP** (mg/l)		
Mean (SE)	0.38 (0.01)	0.66 (0.02)
>10	5.40	14.94
**Albumin** (g/l)		
Mean (SE)	41.88 (0.17)	40.00 (0.17)
<35	2.28	6.50
**HDL**-**cholesterol** (mmol/L)		
Mean (SE)	106.81 (39.29)	94.02 (35.09)
<1.03	22.48	28.38
**Gamma**-**glutamyltransferase** (U/L)		
Mean (SE)	27.85 (0.54)	38.71 (1.28)
>36	16.80	25.23
**Mortality score**		
0	61.60	47.11
1	30.54	34.95
2	7.18	14.35
3	0.65	2.99
4	0.02	0.61
**Cancer**-**specific death** (ICD10: C00-C99)		24.01
**Circulatory death** (ICD10: I00-I99)		41.53

A multivariate Cox proportional hazards model including continuous variables of CRP (mg/L), albumin (g/L), GGT (U/L), and HDL (mmol/L) showed that CRP and GGT were statistically significantly associated with all-cause mortality: HR per one unit increase: 1.19 (95%CI: 1.11, 1.27), 0.95 (0.93, 0.96), 1.31 (1.25, 1.39), and 0.98 (95%CI: 0.91, 1.05), respectively. A log transformation was performed for CRP, GGT, and HDL due to their skewed distributions.

**Figure 1:**
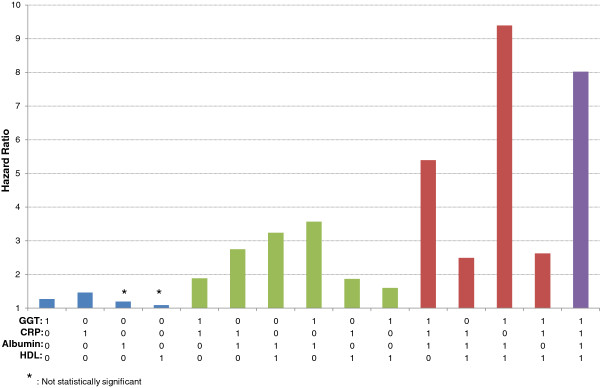
**Hazard ratio for risk of all-cause death by different values of the mortality score.** The model was adjusted for age, gender, poverty to income ratio, race/ethnicity, smoking behavior, alcohol consumption, vigorous physical activity, Charlson Comorbidity Index, and BMI.

When using the mortality score, there was a statistically significant positive association between the score and all-cause mortality as well as cancer and circulatory disease-specific death (e.g. HR for all-cause mortality: 1.21 (95%CI: 1.09, 1.35), 1.92 (95%CI: 1.67, 2.20), 3.38 (95%CI: 2.62, 4.36), and 7.93 (95%CI: 5.77, 10.89) for score=1, 2, 3 and 4 compared to score=0). Similar patterns were found when stratifying by sex and age groups (Table 2 and 3). Figure 1 also shows how each marker of the mortality score contributes to the risk of death. There is no clear pattern by specific biomarkers, but GGT and albumin seem to contribute more than CRP and HDL cholesterol.

**Table 2:** Hazard Ratio (HR) and 95% Confidence Intervals (CI) for risk of all-cause, cancer-specific, and circulatory disease death

	**Total**						**Men**						**Women**					
	**All**-**cause**		**Cancer**		**Circulatory**		**All**-**cause**		**Cancer**		**Circulatory**		**All**-**cause**		**Cancer**		**Circulatory**	
	**HR**	**95%CI**	**HR**	**95%CI**	**HR**	**95%CI**	**HR**	**95%CI**	**HR**	**95**%**CI**	**HR**	**95%CI**	**HR**	**95%CI**	**HR**	**95%CI**	**HR**	**95%CI**
**Score**=**0**	1.00	Ref	1.00	Ref	1.00	Ref	1.00	Ref	1.00	Ref	1.00	Ref	1.00	Ref	1.00	Ref	1.00	Ref
**Score**=**1**	1.21	1.09, 1.35	1.12	0.91, 1.38	1.28	1.08, 1.51	1.16	1.00, 1.34	1.28	1.00, 1.64	1.18	0.97, 1.43	1.25	1.09, 1.43	0.98	0.70, 1.37	1.34	1.06, 1.70
**Score**=**2**	1.92	1.67, 2.20	1.33	0.92, 1.93	2.20	1.85, 2.62	1.64	1.33, 2.02	1.14	0.72, 1.80	1.92	1.55, 2.37	2.40	1.90, 3.04	1.85	0.97, 3.51	2.52	1.82, 3.46
**Score**=**3**	3.38	2.62, 4.36	2.03	1.33, 3.08	3.80	1.82, 6.29	4.13	2.94, 5.80	2.45	1.38, 4.35	3.40	1.74, 6.63	2.43	1.71, 3.46	1.45	0.84, 2.50	2.99	1.71, 7.63
**Score**=**4**	7.93	5.77, 10.89	5.63	3.75, 8.43	8.84	4.78, 16.37	6.13	3.76, 9.97	5.14	1.68, 15.70	7.56	4.00, 14.31	9.80	6.98, 13.75	5.90	3.56, 9.77	10.58	5.01, 22.35
**P for trend**	<0.001		0.007		<0.001		<0.001		0.030		<0.001		<0.001		0.080		<0.001	

All models were adjusted for age, gender, poverty to income ratio, race ethnicity group, smoking behavior, alcohol consumption, vigorous physical activity, BMI, and Charlson Comorbidity Index.

**Table 3:** Age-group specific analysis: Hazard Ratio (HR) and 95% Confidence Intervals (CI) for risk of all cause, cancer-specific, and circulatory disease death

	**Age**-**group** <**65** (**N**=**9873**)						**Age**-**group 65**+ (**N**=**3183**)					
	** All cause**		** Cancer**		** Circulatory**		** All cause**		** Cancer**		** Circulatory**	
	** N**=**875**		** N**=**257**		** N**=**294**		** N**=**2**,**206**		** N**=**396**		** N**=**1094**	
	**HR**	**95%CI**	**HR**	**95%CI**	**HR**	**95%CI**	**HR**	**95%CI**	**HR**	**95%CI**	**HR**	**95%CI**
**Score**=**0**	1.00	Ref	1.00	Ref	1.00	Ref	1.00	Ref	1.00	Ref	1.00	Ref
**Score**=**1**	1.21	0.96, 1.53	1.12	0.78, 1.61	1.51	0.93, 2.45	1.21	1.05, 1.40	1.13	0.87, 1.45	1.21	0.99, 1.47
**Score**=**2**	2.02	1.57, 2.61	1.37	0.74, 2.51	2.75	1.69, 4.49	1.88	1.65, 2.15	1.26	0.94, 1.70	2.00	1.68, 2.38
**Score**=**3**	3.34	2.11, 5.29	0.87	0.37, 2.05	4.15	1.61, 10.70	3.47	2.69, 4.47	3.81	2.38, 6.11	2.79	1.50, 5.18
**Score**=**4**	7.31	2.42, 22.01			4.24	0.94, 19.14	8.27	5.68, 12.05	8.47	5.58, 12.87	9.99	5.52, 18.08
**P for trend**	<0.001		0.362		<0.001		<0.001		<0.001		<0.001	

All models were adjusted for age, gender, poverty to income ratio, race ethnicity group, smoking behavior, alcohol consumption, vigorous physical activity, BMI, and Charlson Comorbidity Index.

To compare the mortality score with the more commonly used CCI, we estimated the association between the CCI and all-cause mortality, which showed analogous risks (HR: 1.06 (95%CI: 0.45, 2.49), 1.55 (95%CI: 0.65, 3.71), and 2.41 (95%CI: 1.00, 5.76) for CCI=2, 3, and 4+ compared to CCI=0; *P* for trend<0.001). Effect modification by CCI for the association between the mortality score and risk of dying was then assessed with stratified analyses by categories of CCI (Table 4). The patterns observed in Table 2 were seen in each category of CCI, even among those with a CCI < 3. For example, among those with CCI =3, the risk of cancer-specific death was 1.10 (95%CI: 0.93, 1.28), 1.81 (95%CI: 1.43, 2.29), 4.67 (95%CI: 3.05, 7.14), and 6.97 (95%CI: 5.32, 9.14) for score=1, 2, 3 and 4 compared to score=0 (Table 4). The univariate association between the mortality score and the CCI did not show a strong correlation between both measurements (correlation coefficient: 0.15; *P*<0.001 and kappa’s coefficient of agreement: 0.01; *P*<0.001).

**Table 4:** Hazard Ratio (HR) and 95% Confidence Intervals (CI) for risk of all-cause, cancer-specific, and circulatory death, stratified by Charlson Comorbidity index

	**Total**						**Men**						**Women**					
	**All**-**cause**		**Cancer**		**Circulatory**		**All**-**cause**		**Cancer**		**Circulatory**		**All**-**cause**		**Cancer**		**Circulatory**	
	**HR**	**95%CI**	**HR**	**95%CI**	**HR**	**95%CI**	**HR**	**95%CI**	**HR**	**95%CI**	**HR**	**95%CI**	**HR**	**95%CI**	**HR**	**95%CI**	**HR**	**95%CI**
**Comorbidity Index** <**3**																		
**Score**=**0**	1.00	Ref	1.00	Ref	1.00	Ref	1.00	Ref	1.00	Ref	1.00	Ref	1.00	Ref	1.00	Ref	1.00	Ref
**Score**=**1**	1.30	1.07, 1.58	0.97	0.70, 1.36	1.47	1.09, 1.98	1.22	0.85, 1.58	1.30	0.91, 1.85	1.12	0.78, 1.60	1.41	1.00, 1.99	0.63	0.29, 1.36	2.15	1.21, 3.72
**Score**=**2**	1.85	1.45, 2.38	1.23	0.68, 2.21	2.73	1.68, 4.43	1.53	1.14, 2.00	0.83	0.50, 1.37	2.25	1.40, 3.62	3.05	2.05, 4.52	2.50	0.93, 6.71	4.30	1.94, 9.53
**Score**=**3**	3.16	2.03, 4.94	0.64	0.16, 2.50	1.06	0.30, 3.75	3.30	1.94, 5.64	0.63	0.12, 3.27	0.87	0.22, 3.44	1.89	0.52, 6.79	0.52	0.06, 4.84	1.32	0.08, 22.13
**Score**=**4**	13.32	6.15, 28.87			17.66	9.86, 31.64	11.27	5.72, 22.22			19.20	9.17, 40.20						
**P for trend**	<0.001		0.844		<0.001		0.002		0.938		0.009		<0.001		0.692		0.001	
**Comorbidity Index** =**3**																		
**Score**=**0**	1.00	Ref	1.00	Ref	1.00	Ref	1.00	Ref	1.00	Ref	1.00	Ref	1.00	Ref	1.00	Ref	1.00	Ref
**Score**=**1**	1.30	1.07, 1.57	1.31	0.87, 1.96	1.15	0.81, 1.62	1.18	0.93, 1.43	1.54	1.00, 2.37	1.43	1.06, 1.92	0.99	0.81, 1.19	0.84	0.43, 1.63	1.08	0.85, 1.39
**Score**=**2**	1.98	1.60, 2.44	1.29	0.80, 2.08	2.18	1.63, 2.90	1.81	1.08, 2.19	1.93	0.97, 3.85	1.35	0.99, 1.86	2.47	1.74, 3.50	1.04	0.44, 2.43	2.26	1.58, 3.23
**Score**=**3**	2.52	1.99, 3.18	3.27	2.07, 5.17	2.38	1.28, 4.41	3.57	3.57, 9.86	4.36	1.86, 10.23	5.96	2.10, 16.92	3.69	1.81, 7.55			6.66	1.97, 22.50
**Score**=**4**	10.22	5.08, 20.58	3.66	1.93, 6.95	16.55	6.66, 41.11	6.23	2.14, 15.81			0.64	0.09, 4.85	9.14	6.71, 12.46	9.36	3.73, 23.52	4.55	1.09, 18.99
**P for trend**	<0.001		0.009		<0.001		<0.001		0.001		0.001		<0.001		0.777		<0.001	
**Comorbidity Index** =**4**																		
**Score**=**0**	1.00	Ref	1.00	Ref	1.00	Ref	1.00	Ref	1.00	Ref	1.00	Ref	1.00	Ref	1.00	Ref	1.00	Ref
**Score**=**1**	1.30	1.07, 1.58	0.97	0.70, 1.36	1.47	1.09, 1.98	1.22	0.85, 1.58	0.93	0.56, 1.56	1.07	0.71, 1.62	1.41	1.11, 1.78	1.72	0.88, 3.33	1.25	0.84, 1.86
**Score**=**2**	1.85	1.45, 2.38	1.23	0.68, 2.21	2.73	1.68, 4.43	1.53	1.14, 2.00	0.68	0.35, 1.35	2.04	1.29, 3.24	2.16	1.57, 2.98	2.06	0.98, 4.37	2.28	1.42, 3.65
**Score**=**3**	3.16	2.03, 4.94	0.64	0.16, 2.50	1.06	0.30, 3.75	3.30	1.94, 5.64	3.51	1.66, 7.44	3.85	1.79, 8.29	1.60	1.02, 2.53	3.04	1.41, 6.53	0.71	0.23, 2.20
**Score**=**4**	13.32	6.15, 28.87			17.66	9.86, 31.64	11.27	5.72, 22.22			10.76	5.29, 21.89	14.44	6.11, 34.15	5.85	2.61, 13.09	26.72	7.81, 91.37
**P for trend**	<0.001		0.003		<0.001		<0.001		0.965		<0.001		<0.001		0.003		0.002	

All models were adjusted for age, gender, poverty to income ratio, race ethnicity group, smoking behavior, alcohol consumption, vigorous physical activity, and BMI.

A stratified analysis by race/ethnicity showed that the score predicted mortality in a similar way for non-Hispanic white, non-Hispanic blacks, and Mexican American (Table 5). The results for cancer and circulatory death were also comparable with the findings in Table 2.

**Table 5:** Hazard Ratio (HR) and 95% Confidence Intervals (CI) for risk of all-cause, cancer-specific, and circulatory death, stratified by race/ethnicity

	**Total**					
	** All**-**cause**		** Cancer**		** Circulatory**	
	**HR**	**95%CI**	**HR**	**95%CI**	**HR**	**95%CI**
**Non**-**Hispanic white**						
**Score**=**0**	1.00	Ref	1.00	Ref	1.00	Ref
**Score**=**1**	1.17	1.04, 1.31	1.03	0.83, 1.28	1.31	1.09, 1.57
**Score**=**2**	1.98	1.64, 2.39	1.25	0.88, 1.99	2.44	1.70, 3.03
**Score**=**3**	3.11	2.31, 4.19	1.39	0.88, 2.18	3.43	1.62, 7.26
**Score**=**4**	9.51	6.40, 14.14	7.80	5.09, 11.95	10.50	5.38, 20.47
**P for trend**	<0.001		0.154		<0.001	
**Non**-**Hispanic black**						
**Score**=**0**	1.00	Ref	1.00	Ref	1.00	Ref
**Score**=**1**	1.45	1.23, 1.71	1.53	1.09, 2.15	1.30	1.04, 1.61
**Score**=**2**	1.77	1.38, 2.26	1.42	0.89, 2.29	1.49	1.02, 2.16
**Score**=**3**	4.86	3.38, 6.99	6.76	3.64, 12.55	3.19	1.77, 5.76
**Score**=**4**	3.76	1.41, 10.04	4.39	0.75, 25.52	2.46	0.46, 13.21
**P for trend**	<0.001		<0.001		0.001	
**Mexican American**						
**Score**=**0**	1.00	Ref	1.00	Ref	1.00	Ref
**Score**=**1**	1.04	0.85, 1.27	0.55	0.26, 1.14	1.15	0.80, 1.65
**Score**=**2**	1.31	0.87, 1.98	0.47	1.19, 1.16	1.35	0.86, 2.10
**Score**=**3**	3.65	1.93, 6.89	3.40	0.95, 12.11	2.72	1.34, 5.52
**Score**=**4**	11.79	5.85, 23.78			14.45	6.17, 33.84
**P for trend**	0.002		0.356		0.007	

All models were adjusted for age, gender, poverty to income ratio, smoking behavior, alcohol consumption, vigorous physical activity, Charlson Comorbidity Index, and BMI.

Excluding those with follow-up of <1 year, as part of a sensitivity analysis, showed similar patterns to those observed in Table 2 (results not shown). For example, the HRs for all-cause mortality increased with values of the mortality score: 1.20 (95%CI: 1. 07, 1.34), 1.81 (95%CI: 1.58, 2.08), 2.99 (95%CI: 2.33, 3.84), and 5.55 (95%CI: 4.32, 7.14), for score=1, 2, 3 and 4 compared to score=0. Similar observations were made when excluding those with follow-up <3 or <5 years (results not shown).

## Discussion

Using NHANES-III data we could replicate the results of the Swedish AMORIS study [1]. The combination of serum levels of CRP, albumin, GGT, and HDL was positively associated with mortality and was even predictive in patients with low comorbidity based on CCI. Furthermore, the association between the score and mortality was found to be similar among non-Hispanic whites, non-Hispanic blacks, and Mexican Americans.

The three suggested mechanisms, inflammation, liver dysfunction, and lipid metabolism, have been associated with early death and comorbidities in several different research settings. The inflammation-based Glasgow Prognostic Score (GPS), based on serum levels of CRP and albumin, has repeatedly been shown to be a predictor of survival, independent of tumor stage, performance status and treatment [3,18-20]. In a study of 540 cancer patients, increasing GPS correlated with more aggressive tumor biology in terms of tumor size, presence of lymph node metastasis, and higher tumor recurrence rate [20]. In addition to being a marker of liver dysfunction, high levels of GGT (>28 U/L) have been found to be positively associated with risk of all cause mortality and risk of developing cancer [21,22]. It is also thought that persistent production of reactive oxygen stress following increased GGT expression in tumor cells contributes to genetic instability and tumor progression [19]. Finally, HDL cholesterol, as a component of the metabolic syndrome, has been extensively studied in relation to early death and comorbidities [12,23]. Apart from its link with the lipid metabolism, HDL is also associated with inflammation [6,23,24]. More specifically, HDL has been linked with pro-inflammatory cytokines such as tumor necrosis factor-α (TNF-α) [25]. It has been shown that HDL reduces free TNF-α resulting in reduced tissue damage, reduced infiltration of macrophages and neutrophils, and potential attenuated tumor formation [26]. A prospective study of 989 persons aged 65 and over, showed that high levels of HDL were significantly associated with low total risk of death in men [27].

As in the Swedish AMORIS study, the HRs found using data from NHANES III were also of a magnitude that may be of clinical relevance. Even though Figure 1 did not show a clear pattern by specific markers of the mortality score, albumin and GGT seemed to contribute slightly more to the prediction of death than the other markers. It is important to note that these results are slightly different from our analysis based on continuous levels of the four studied biomarkers, which is likely explained by the more medically relevant cut-offs used in our mortality score. The findings in Figure 1 suggest that markers of inflammation and reactive oxygen species are more strongly associated with risk of death. Because of limited sample size, it was not possible to perform identical age-stratified analysis so that we could not identify the predictive strength of the score in those aged 50–65. In our analysis, the score was found to be equally predictive of death among those younger and older than 65. Interestingly, we also replicated the findings stratified by categories of the CCI. Among those with CCI<3, there was a positive trend between the mortality score and all-cause mortality as well as circulatory disease-specific death. The association was not statistically significant with cancer-specific death; this may be due to the comparably small number of cancer deaths (24 versus 42%). Nevertheless, the results by categories of CCI indicate that the mortality score is predictive for mortality over and above the prediction by CCI.

In our study we could also evaluate the mortality score in different ethnic groups. A study evaluating the clinical utility of the triglycerides-to-HDL ratio showed that this ratio may identify insulin-resistance in Aboriginals, Chinese, and Europeans, but not South Asians, thus reflecting possible ethnic differences in normal lipid levels [28]. Also, levels of albumin also differed by ethnicity. Among UK resident patients of white European or south Asian ethnicity with type 2 diabetes mellitus there were significant differences in microalbuminuria [29]. In addition, the association between CRP and disease outcome appeared to be influenced by ethnicity. In a multi-ethnic cohort including 2362 Caucasians, 1601 African Americans, 1353 Hispanics, and 751 Chinese, CRP was only associated with the risk of circulatory disease in Caucasians [30]. This variation by ethnicity was smaller for GGT. In another study based on NHANES III, the correlation between GGT and risk of hypertension was consistent by race-ethnicity [31]. In our study there was also no effect-modification by race-ethnicity; the mortality score seemed to predict all cause, cancer-specific and circulatory death in a consistent way across ethnic groups. This may suggest that a combination of these four biomarkers is more general and less susceptible to subgroup differences.

This study has several strengths including its generalizibility following the use of nationally representative data. Therefore it was also possible in our analysis to perform a stratified analysis by race/ethnicity. We were able to adjust for many potential confounding factors and examine interactions by age and the CCI. In a sensitivity analysis, we used different medical cut-offs by sex for HDL and GGT, but this did not materially alter our findings (results not shown). Another limitation of this study and the mortality score is that it relies on one single measurement so that it may be prone to measurement error and within-person variation. Repeated measurements may strengthen the accuracy of the score. In addition, it is important to note that the current mortality score only represents a selection of mechanisms and markers associated with early death. It is possible that other markers such as hypertension or the ratio of total to HDL cholesterol may also be good components of a mortality score, however we believe that the current mortality score already represents three important mechanisms associated with early death. A next step in the process of potentially taking this score in clinic is the performance of sensitivity and specific tests in order to determine the most predictive cut-offs for the different biomarkers used.

## Conclusion

The combination of four biomarkers predicted all-cause mortality as well as cancer and circulatory disease-specific death in NHANES III. This is in agreement with a Swedish study and was consistent across different ethnic groups. This trans-ethnic validity and the positive association for all categories of the CCI suggests that this score may help clinicians making decisions in prevention and disease management.

## Competing interests

The authors declare that they have no competing interests.

## Authors’ contributions

MVH designed the study, conducted the statistical analyses, interpreted the data, and drafted the manuscript. ME, DF, and SR assisted in data interpretation and critically reviewed the manuscript. SR supervised the study. All authors read and approved the final manuscript.

## Pre-publication history

The pre-publication history for this paper can be accessed here:

http://www.biomedcentral.com/1471-2458/12/895/prepub

## References

[B1] Van Hemelrijck M Harari D Garmo H Hammar N Walldius G Lambe M Biomarker-based score to predict mortality in persons aged 50 years and older: a new approach in the Swedish AMORIS study International Journal of Molecular Epidemiology and Genetics 2012 PMC3316450 22493753

[B2] Fulop T Larbi A Witkowski JM McElhaney J Loeb M Mitnitski A Aging, frailty and age-related diseases Biogerontology 2010 PubMed ID: 20559726 10.1007/s10522-010-9287-2 20559726

[B3] McMillan DC An inflammation-based prognostic score and its role in the nutrition-based management of patients with cancer Proc Nutr Soc 2008 67 3 257 262 10.1017/S0029665108007131 18452641

[B4] Targher G Elevated serum gamma-glutamyltransferase activity is associated with increased risk of mortality, incident type 2 diabetes, cardiovascular events, chronic kidney disease and cancer - a narrative review Clin Chem Lab Med 2010 48 2 147 157 19943812 10.1515/CCLM.2010.031

[B5] Landi F Russo A Pahor M Capoluongo E Liperoti R Cesari M Serum high-density lipoprotein cholesterol levels and mortality in frail, community-living elderly Gerontology 2008 54 2 71 78 10.1159/000111381 18025809

[B6] Kaysen GA Biochemistry and biomarkers of inflamed patients: why look, what to assess Clin J Am Soc Nephrol 2009 4 Suppl 1 S56 S63 19996007 10.2215/CJN.03090509

[B7] Pallis AG Fortpied C Wedding U Van Nes MC Penninckx B Ring A EORTC elderly task force position paper: approach to the older cancer patient Eur J Cancer 2010 46 9 1502 1513 10.1016/j.ejca.2010.02.022 20227872

[B8] National Center for Health Statistics Plan and operation of the Third National Health and Nutrition Examination Survey, 1988–94. Series 1: programs and collection procedures Vital Health Stat 1994 1 32 1 407 7975354

[B9] NHANES III Linked Mortality File [database on the Internet]2009 2011 http://www.cdc.gov/nchs/data_access/data_linkage/mortality/nhanes3_linkage.htm

[B10] Gunter E Lewis B Koncikowski S Laboratory procedures used for the Third National Health and Nutrition Survey (NHANES III) 1998 Atlanta, GA: Centers for Disease Control and Prevention, Public Health Service, US Department of Health and Human Services1996, National Center for Environmental Health

[B11] Goldfarb-Rumyantzev AS Rout P Sandhu GS Khattak M Tang H Barenbaum A Association between social adaptability index and survival of patients with chronic kidney disease Nephrol Dial Transplant 2010 25 11 3672 3681 10.1093/ndt/gfq177 20353959

[B12] Executive Summary of The Third Report of The National Cholesterol Education Program (NCEP) Expert Panel on Detection, Evaluation, And Treatment of High Blood Cholesterol In Adults (Adult Treatment Panel III) JAMA 2001 285 19 2486 2497 10.1001/jama.285.19.2486 11368702

[B13] Strasak AM Pfeiffer RM Klenk J Hilbe W Oberaigner W Gregory M Prospective study of the association of gamma-glutamyltransferase with cancer incidence in women Int J Cancer 2008 123 8 1902 1906 10.1002/ijc.23714 18688855

[B14] Solhpour A Parkhideh S Sarrafzadegan N Asgary S Williams K Jungner I Levels of lipids and apolipoproteins in three cultures Atherosclerosis 2009 207 1 200 207 10.1016/j.atherosclerosis.2009.09.003 19766218

[B15] Bateman BT Shaw KM Kuklina EV Callaghan WM Seely EW Hernandez-Diaz S Hypertension in women of reproductive age in the United States: NHANES 1999–2008 PLoS One 2012 7 4 e36171 10.1371/journal.pone.0036171 22558371 PMC3340351

[B16] Hunt KJ Gebregziabher M Egede LE Racial and ethnic differences in cardio-metabolic risk in individuals with undiagnosed diabetes: national health and nutrition examination survey 1999–2008 J Gen Intern Med 2012 27 8 893 900 10.1007/s11606-012-2023-7 22415867 PMC3403154

[B17] Redmond N Baer HJ Hicks LS Health behaviors and racial disparity in blood pressure control in the national health and nutrition examination survey Hypertension 2011 57 3 383 389 10.1161/HYPERTENSIONAHA.110.161950 21300667 PMC3048351

[B18] Proctor MJ Morrison DS Talwar D Balmer SM Fletcher CD O'Reilly DS A comparison of inflammation-based prognostic scores in patients with cancer. A Glasgow Inflammation Outcome Study Eur J Cancer 2011 47 17 2633 2641 10.1016/j.ejca.2011.03.028 21724383

[B19] Crumley AB Stuart RC McKernan M McDonald AC McMillan DC Comparison of an inflammation-based prognostic score (GPS) with performance status (ECOG-ps) in patients receiving palliative chemotherapy for gastroesophageal cancer J Gastroenterol Hepatol 2008 23 8 Pt 2 e325 e329 17645468 10.1111/j.1440-1746.2007.05105.x

[B20] Vashist YK Loos J Dedow J Tachezy M Uzunoglu G Kutup A Glasgow Prognostic Score is a predictor of perioperative and long-term outcome in patients with only surgically treated esophageal cancer Ann Surg Oncol 2011 18 4 1130 1138 10.1245/s10434-010-1383-7 20981494

[B21] Ulmer H Kelleher C Diem G Concin H Why Eve is not Adam: prospective follow-up in 149650 women and men of cholesterol and other risk factors related to cardiovascular and all-cause mortality J Womens Health (Larchmt) 2004 13 1 41 53 10.1089/154099904322836447 15006277

[B22] Van Hemelrijck M Jassem W Walldius G Fentiman IS Hammar N Lambe M Gamma-glutamyltransferase and risk of cancer in a cohort of 545,460 persons - the Swedish AMORIS study Eur J Cancer 2011 47 13 2033 2041 10.1016/j.ejca.2011.03.010 21486691

[B23] Van Hemelrijck M Walldius G Jungner I Hammar N Garmo H Binda E Low levels of apolipoprotein A-I and HDL are associated with risk of prostate cancer in the Swedish AMORIS study Cancer Causes Control 2011 10.1007/s10552-011-9774-z 21562751

[B24] Jacobs EJ Gapstur SM Cholesterol and cancer: answers and new questions Cancer Epidemiol Biomarkers Prev 2009 18 11 2805 2806 10.1158/1055-9965.EPI-09-1027 19887583

[B25] Simpson DC Kabyemela E Muehlenbachs A Ogata Y Mutabingwa TK Duffy PE Plasma levels of apolipoprotein A1 in malaria-exposed primigravidae are associated with severe anemia PLoS One 2010 5 1 e8822 10.1371/journal.pone.0008822 20098675 PMC2809092

[B26] Calabresi L Rossoni G Gomaraschi M Sisto F Berti F Franceschini G High-density lipoproteins protect isolated rat hearts from ischemia-reperfusion injury by reducing cardiac tumor necrosis factor-alpha content and enhancing prostaglandin release Circ Res 2003 92 3 330 337 10.1161/01.RES.0000054201.60308.1A 12595346

[B27] Chyou PH Eaker ED Serum cholesterol concentrations and all-cause mortality in older people Age Ageing 2000 29 1 69 74 10.1093/ageing/29.1.69 10690699

[B28] Gasevic D Frohlich J Mancini GB Lear SA 2011 Metabolism: The association between triglyceride to high-density-lipoprotein cholesterol ratio and insulin resistance in a multiethnic primary prevention cohort 10.1016/j.metabol.2011.09.009 22075272

[B29] Raymond NT Paul O'Hare J Bellary S Kumar S Jones A Barnett AH Comparative risk of microalbuminuria and proteinuria in UK residents of south Asian and white European ethnic background with type 2 diabetes: a report from UKADS Curr Med Res Opin 2011 27 Suppl 3 47 55 22106977 10.1185/03007995.2011.614937

[B30] Veeranna V Zalawadiya SK Niraj A Kumar A Ference B Afonso L Association of novel biomarkers with future cardiovascular events is influenced by ethnicity: Results from a multi-ethnic cohort Int J Cardiol 2012 10 PubMed ID: 22240756 10.1016/j.ijcard.2011.11.034 22240756

[B31] Shankar A Li J Association between serum gamma-glutamyltransferase level and prehypertension among US adults Circ J 2007 71 10 1567 1572 10.1253/circj.71.1567 17895553

